# Increased carotid artery stiffness after preeclampsia in a cross‐sectional study of postpartum women

**DOI:** 10.14814/phy2.15276

**Published:** 2022-04-19

**Authors:** Logan C. Barr, Julia E. Herr, Marie‐France Hétu, Graeme N. Smith, Amer M. Johri

**Affiliations:** ^1^ Department of Biomedical and Molecular Sciences Queen’s University Kingston Ontario Canada; ^2^ Cardiovascular Imaging Network at Queen’s Department of Medicine Queen’s University Kingston Ontario Canada; ^3^ Department of Obstetrics & Gynaecology Queen’s University Kingston Ontario Canada

**Keywords:** carotid arteries, postpartum period, preeclampsia, vascular stiffness

## Abstract

Preeclampsia (PE) is a hypertensive obstetrical complication associated with increased cardiovascular disease risk. Carotid artery functional assessments allow for identification of subclinical vascular dysfunction. This cross‐sectional study measured carotid artery functional indices in healthy women with a recent pregnancy complicated by PE, versus women with a prior uncomplicated pregnancy. Women with a history of PE (*N* = 30) or an uncomplicated pregnancy (*N* = 30), were recruited between 6 months and 5 years postpartum. Left and right carotid artery ultrasound measured carotid intima media thickness, plaque burden, peak systolic velocity, end diastolic flow velocity and carotid far‐wall circumferential strain (FWCS). Carotid FWCS is inversely related to vessel stiffness, where a decrease in FWCS indicates increased vessel stiffness. Right‐side FWCS did not differ between women with a history of PE versus normotensive pregnancy. Left carotid artery FWCS was lower in formerly preeclamptic women after adjustment for diameter, pulse pressure, and heart rate compared to women following an uncomplicated pregnancy (3.35 ± 1.08 × 10^−3^ vs. 4.46 ± 1.40 × 10^−3^; *p* = 0.003). Those with prior severe PE had the greatest decrease in FWCS adjusted to diameter, pulse pressure, and heart rate compared to healthy controls (*p* = 0.02). Adjusted FWCS and total serum cholesterol were independent indicators of PE history when present in a logistic regression model with confounding variables including age, body mass index, and resting blood pressure. Further investigation is needed to elucidate if FWCS can be used as a risk stratification tool for future cardiovascular disease following a pregnancy complicated by PE. A history of PE is associated with decreased left FWCS (increased left carotid artery stiffness).

## Graphical abstract

Carotid artery strain is decreased in woman following a pregnancy complicated by preeclampsia, compared to women with uncomplicated pregnancy. No differences were observed with respect to plaque burden, blood flow velocity, and carotid intima‐media thickness. Created with Biorender (www.biorender.com).

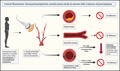

**Highlights**

Carotid artery function was assessed after preeclampsia or uncomplicated pregnancy. Left carotid far‐wall circumferential strain, which is inversely correlated with carotid stiffness, was lower after severe preeclampsia. Carotid artery stiffness after severe preeclampsia may inform future disease risk.

## 1 INTRODUCTION

Preeclampsia (PE) is amongst the most serious and poorly understood risks to maternal health in pregnancy. It is a multi‐factorial condition which may arise from defective placentation, maternal immune hyperreactivity, or pre‐existing cardiometabolic risk (Redman et al., 2014). PE is characterized by new‐onset hypertension, proteinuria, and other end organ effects in the late stages of gestation. Current therapeutic strategies for PE are limited to blood pressure management, seizure prevention, and delivery of the fetoplacental unit, with ongoing research about prophylaxis with low‐dose aspirin (Villar et al., 2004). Women with a history of PE are at a higher risk for future hypertension, diabetes, and cardiovascular disease (Smith et al., 2019).

Maternal vascular aberrations, thought to be secondary to oxidative stress, immune activation, and placental dysregulation are all hallmarks of PE. Cardiovascular dysfunction during PE has been observed in the retinal microcirculation, conduit vasculature and in large elastic vessels such as the aorta and carotid arteries (Brückmann et al., 2015; Robb et al., 2009; Takase et al., 2003; Torrado et al., 2015). Therefore, vascular dysfunction associated with PE in pregnancy may serve as a catalyst for future maternal cardiovascular risks. However, accounts of vascular dysfunction following PE are sparse and inconsistent (Kvehaugen et al., 2011; Lampinen et al., 2006; Murphy et al., 2014).

Changes in arterial functional risk indicators such as vessel stiffness, plaque area and height, and pulse wave velocity are harbingers of incipient cardiovascular disease (CVD). Assessment of large vessels, such as the carotid artery, allows clinicians to observe pre‐clinical vascular dysfunction, providing an early opportunity for medical intervention before onset of disease. Carotid artery systolic and end diastolic velocimetry provide an index of internal carotid artery stenosis (Alexandrov et al., 1997; Costa et al., 1997). Intima‐media thickening is a surrogate measure of vascular remodeling to hypertensive stress or incipient atherosclerotic processes (Bots et al., 2002). Carotid artery stiffness reflects a loss of vessel elasticity that may stem from endothelial adaptation to cardiovascular risk factors (Bots et al., 2002). Carotid artery stiffening may increase pulsatility and central blood pressure, which is thought to increase the risk of cerebrovascular events (Van Sloten et al., 2015). Speckle tracking of circumferential deformation in the common carotid artery (far‐wall circumferential strain) is a novel, reliable method of measuring carotid artery stiffness (Patton et al., 2018; Yang et al., 2014)

Assessment of vascular alterations following PE may help to clarify downstream cardiovascular risk associated with the condition and may aid in the development of risk stratification tools that could identify women at risk of developing CVD. Additionally, profiling of vascular functional changes after PE may pinpoint therapeutic targets that could eventually give rise to clinical interventions. Therefore, we undertook an exploratory study in postpartum women to test our hypothesis that carotid arterial intima‐media thickness, plaque burden, blood velocity, and strain are altered in women with a history of PE compared to women following uncomplicated pregnancies.

## 2 MATERIALS AND METHODS

Ethics statement: This was a cross‐sectional study conducted in the Kingston, Ontario area region between 2017 and 2019. This study was approved by the Queen’s University Health Sciences and Affiliated Teaching Hospitals Research Ethics Board (#6016991). All participants provided written informed consent for this study. All components of the investigation conformed to the principles outlined in the Declaration of Helsinki.

History of PE participants: Women were recruited by convenience sampling who had received a post‐partum clinic visit for cardiovascular risk screening at the Maternal Health Clinic at the Kingston Health Sciences Center (Smith, 2015) within the past 5 years. The basis of the initial visit was for a confirmed diagnosis of PE by American College of Obstetricians and Gynecologists criteria, but PE status was confirmed with a physician prior to enrolment (ACOG, 2020). PE was additionally classified as “PE with severe features” based on blood pressure >160/100 mmHg, preterm gestational age at delivery <37 weeks; thrombocytopenia, visual disturbances and severe upper right quadrant pain (American College of Obstetricians & Gynecologists, 2013). All eligible case participants developed PE in their most recent pregnancy. Inclusion criteria included: maternal age 18–40 years, between 6 months and 5 years postpartum, and prior singleton index pregnancy. Exclusion criteria included: history of cardiovascular or autoimmune disease prior to pregnancy, or a current course of antihypertensive, anti‐platelet, or cholesterol‐lowering medications (Barr et al., 2021). Uncomplicated participants followed the same inclusion criteria and were recruited by convenience sampling through a combination of community postings, advertisements at Kingston Health Sciences Center and prior research participation.

Participants were instructed to abstain from caffeine on the day of their appointment, but to otherwise follow their normal diet and fluid intake. Demographic and health history information, followed by height, weight, and waist circumference were collected. Blood pressure was obtained as an average of 5 automated measurements (BPTRU‐BPM200, BPTru, Canada). To assess the effect of modifiable risk factors on cardiovascular function, participants’ 30‐year full Framingham risk for cardiovascular disease was calculated (Pencina et al., 2009). This also yields an optimal score for each participant. The difference between a participant’s optimal and actual score was used to classify their 30‐year risk level as either suboptimal (SUB) or optimal (OPT). Additionally, participants were given a requisition for fasted bloodwork at a later date which assessed factors such as fasting glucose, lipids, and high sensitivity C‐reactive protein. Urine was obtained for microalbumin: creatinine ratio determination. In the final dataset, comparisons were drawn between PE and CTRL participants for all demographic, anthropometric, and cardiometabolic factors.

Carotid ultrasound was performed using a Vivid E9 echocardiography system (GE Healthcare, Mississauga, Canada), equipped with a 9 L linear transducer and electrocardiogram and ECG leads. Scans were done on both the left and right carotid arteries. All scans and calculations were performed by trained research staff, blinded to participants’ prior obstetrical history. Variables assessed via ultrasound included carotid intima media thickness (CIMT), plaque burden (plaque height and area), blood flow velocity, and carotid strain. Measurement of CIMT, an established marker of future cardiovascular risk, was conducted in the distal common carotid artery. Plaque burden was measured as the maximum plaque height and the total plaque area of both sides (sum of all apparent carotid plaque) in the carotid bulb and internal carotid artery (Johri et al., 2016). Plaque was defined using the Mannheim plaque consensus, as a focal structure protruding into the arterial lumen by at least 50% of the surrounding intima‐media thickness or measuring ≥1.5 mm in thickness from the vessel wall (Touboul et al., 2012). Doppler assessment of peak systolic blood flow velocity (PSV) and end diastolic velocity was performed in both the common (CCA) and internal (ICA) carotid arteries.

Carotid circumferential strain, a novel biomarker inversely related to vessel stiffness, was measured by speckle tracking in the short axis of the common carotid artery (Patton et al., 2018; Yang et al., 2014). Far‐wall circumferential strain (FWCS) was measured for 3 consecutive cardiac cycles for each side and the mean FWCS calculated using GE software (GE EchoPAC v201; GE Vingmed Ultrasound, Norway). The FWCS was indexed for diameter, pulse pressure (PP = systolic‐diastolic), and heart rate (HR) [(mean FWCS/diameter)/(PP*HR)]. In arterial assessment, a lower positive value of strain (reduction) corresponds to increased stiffness (Yang et al., 2014).

Statistical analyses were conducted using JMP®12.0.1 software (SAS Institute Inc., Cary, NC, USA). The Fisher’s exact test was used to compare nominal variables, and the Wilcoxon–Mann–Whitney two‐sample test was used for continuous variables. Comparisons between groups were drawn for each health variable obtained in the demographic, health history, and ultrasound component of the study visit. A Steel–Dwass all pairs test for nonparametric data was used to assess differences between uncomplicated controls and those with prior mild or severe PE, corrected for three comparisons between groups. We used logistic regression model with a backward selection criterion of *p* < 0.25 to select for independent factors associated with PE. The model included age (years), body mass index (kg/m2), waist circumference (cm), systolic blood pressure (mmHg), diastolic blood pressure (mmHg), ever smoked, hypertension, cholesterol (mmol/L), and adjusted FWCS for assessing correlations to PE. Additionally, we constructed another logistic regression model with a backwards selection criterion of *p* < 0.025 to identify independent factors that could predict adjusted FWCS. As a post‐hoc analysis, receiver operating characteristics (ROC) curves were used to determine the optimal threshold values of adjusted carotid strain for detecting PE. A *p* < 0.05 was deemed statistically significant.

## 3 RESULTS

### 3.1 Sample population characteristics

**FIGURE 1:**
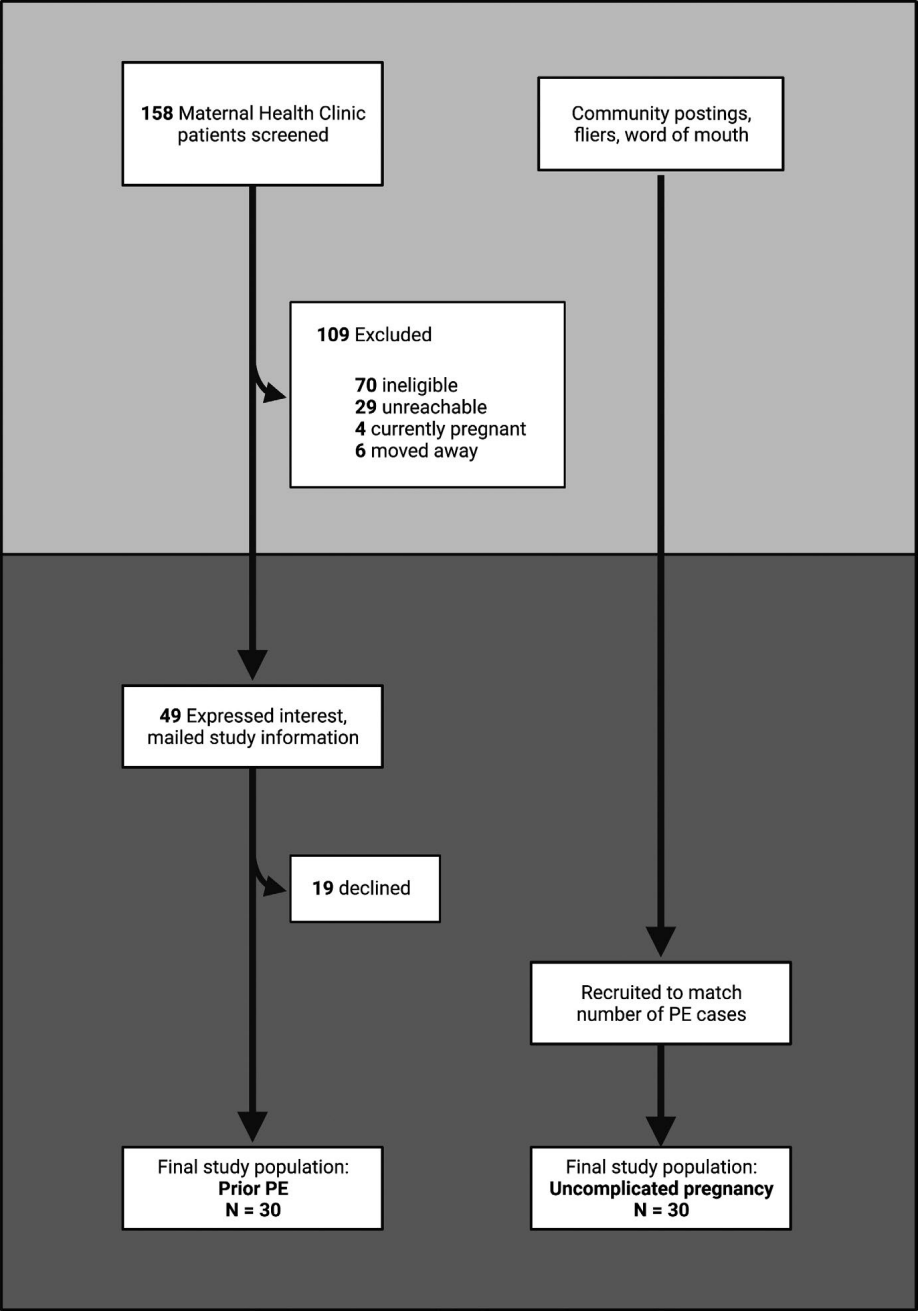
Recruitment scheme for final study population. Formerly preeclamptic women were identified through a postpartum maternal clinic, while control participants volunteered through community postings and word‐of‐mouth

A total of 158 women who had previously consented to research participation, and who had a history of PE, were identified from chart review at the clinic. Of these, 70 participants were ineligible on the basis of age, comorbid diabetes mellitus in pregnancy, personal health history or multi‐fetus gestation; 29 were unreachable by phone; 4 were pregnant or had recently delivered; and 6 had moved away since their Maternal Health Clinic appointment. The remaining 47 women were emailed study information, of which 30 were enrolled into the study (PE group). In parallel, 30 healthy controls (CTRL) were enrolled into the study, giving a total of 60 participants (Figure 1). All 60 participants had complete clinical and vascular assessments.

On average, PE participants were younger than the comparison group (*p* = 0.03) but, had a higher body mass index (BMI) (30.7 ± 7.74 vs. 25.8 ± 4.92; *p =* 0.01) and higher systolic (116.0 ± 13.7 mmHg vs. 107.8 ± 8.51 mmHg; *p* = 0.01) and diastolic (76.9 ± 8.91 mmHg vs. 71.1 ± 6.10 mmHg; *p* = 0.009) blood pressures (Table 1). Additionally, PE participants had a larger waist circumference on average (92.4 ± 16.0 cm) compared to CTRL participants (83.4 ± 11.8; *p* = 0.04) and were more likely to have had a history of smoking (37% vs. 13%; *p* = 0.04) and to currently have hypertension (23% vs. 3%; *p* = 0.03) as defined by the International Diabetes Federation criterion of ≥130/85 mmHg (Alberti et al., 2009). Consistent with the clinical course of PE, participants in the PE group delivered the index pregnancy earlier than their uncomplicated counterparts (36.4 ± 2.81 weeks vs. 39.6 ± 1.15 weeks; *p* < 0.0001). After a blinded chart review, 16 women with PE were identified as having had disease with severe features, while 14 did not have severe features. This is higher than the distribution in the general population, where severe features in PE are less common (Lisonkova & Joseph, 2013). Additionally, four women in the case group (3 non‐severe, 1 severe) had recurrent PE in previous pregnancies.

**TABLE 1:** Sample population characteristics

Variable	Preeclampsia (*N* = 30)	Control (*N* = 30)	*p*‐value
Age (years)	32.0 ± 3.87	34.3 ± 3.57	0.03*
Ethnicity (Caucasian), *n* (%)	27 (90)	29 (97)	0.94
Body mass index (kg/m^2^)	30.7 ± 7.74	25.8 ± 4.92	0.01*
WC (cm)	92.4 ± 16.0	83.4 ± 11.8	0.04*
Systolic blood pressure (mmHg)	116.0 ± 13.7	107.8 ± 8.51	0.01*
Diastolic blood pressure (mmHg)	76.9 ± 8.91	71.1 ± 6.10	0.009*
Heart rate (beats per minute)	69.0 ± 9.15	66.4 ± 8.92	0.33
Pregnancy history			
Time postpartum (weeks)	91.1 ± 70.5	121.1 ± 69.2	0.11
Gestational age at delivery (weeks)	36.4 ± 2.81	39.6 ± 1.15	<0.0001*
Cardiac risk factors			
BMI >30 (kg/m^2^), *N* (%)	15 (50)	7 (23)	0.03*
Currently smoking, *N* (%)	1 (3)	0 (0)	0.5
Ever smoked, *N* (%)	11 (37)	4 (13)	0.04*
Hypertension, *N* (%)	7 (23)	1 (3)	0.03*
30‐year risk score (SUB), *N* (%)	22 (73)	15 (50)	0.06

Data presented as mean ± standard deviation.

Abbreviation: BMI, body mass index; BP, blood pressure; SUB, suboptimal; WC, waist circumference.

* indicates significant (*p* < 0.05) difference as assessed by Fisher’s exact test or Wilcoxon–Mann–Whitney two‐sample test.

### 3.2 Bloodwork and urinalysis

Participants left the research visit with a requisition for bloodwork at a community facility. Adherence to this component of the study was low among CTRL participants. Bloodwork results were assessed on a per‐protocol basis. Of the present study population, 25 PE and 17 CTRL participants underwent bloodwork and urinalysis at the community laboratory (Table 2). Study groups did not differ with respect to serum/urine creatinine measurements; urinary microalbumin; glomerular filtration rate; serum triglycerides, high‐ and low‐density lipoprotein; serum glucose; or serum C‐reactive peptide. However, PE participants had a higher serum total cholesterol level compared to the comparison group (4.82 ± 0.97 vs. 4.25 ± 0.65; *p* = 0.04).

**TABLE 2:** Bloodwork and urinalysis results

Variable	Preeclampsia (*N* = 25)	Control (*N* = 17)	*p*‐value
Serum creatinine (µmol/L)	62.24 ± 18.8	67.65 ± 10.7	0.51
Glomerular filtration rate (mL/min/1.73 m^2^)	103.9 ± 15.5	101.1 ± 14.8	0.58
Urinary microalbumin (mg/L)	13.47 ± 10.10	9.08 ± 6.55	0.19
Urinary creatinine (mmol/L)	14.01 ± 6.12	14.56 ± 6.92	0.8
Urinary albumin/creatinine (mg/mmol)	3.31 ± 11.5	2.00 ± 5.99	0.61
Serum total cholesterol (mmol/L)	4.82 ± 0.97	4.25 ± 0.65	0.04*
Serum triglycerides (mmol/L)	1.17 ± 0.73	0.86 ± 0.48	0.13
Serum high‐density lipoprotein (mmol/L)	1.57 ± 0.47	1.61 ± 0.42	0.76
Serum low‐density lipoprotein (mmol/L)	2.72 ± 0.80	2.32 ± 0.48	0.07
Serum glucose (mmol/L)	4.76 ± 0.39	4.76 ± 0.44	0.97
Serum C‐reactive peptide (mg/L)	3.31 ± 3.97	2.53 ± 2.8	0.23

Data presented as mean ± standard deviation. µmol, micro‐moles; mL, milliliters; mg, milligrams; mmol, millimoles, L, liter.

* indicates significant (*p* < 0.05) difference as assessed by Fisher’s exact test or Wilcoxon–Mann–Whitney two‐sample test.

### 3.3 Carotid artery measurements

In our assessment of traditional carotid ultrasound atherosclerotic imaging markers, there were no differences between PE versus CTRL groups (Table 3). There was no difference in mean CIMT between PE and CTRL. However, the left CIMT was significantly thicker than the right CIMT in PE women (CIMT mean difference = 0.032, CI 0.009‐0.06; *p* = 0.01). This was not observed in the CTRL group (CIMT mean difference = 0.016, 95% CI −0.006–0.038; *p* = 0.08). Common carotid artery diameter did not vary between CTRL and PE individuals. Carotid artery plaque was detected in 5 individuals (3 CTRL, 2 PE), but no difference in plaque height or area was found between groups. Carotid blood flow velocimetry, PSV, EDV, and ratio were within accepted normal range, and did not differ between groups (Grant et al., 2003).

**TABLE 3:** Carotid artery measurements

Variable	Preeclampsia (*N* = 30)		Control (*N* = 30)		*p*‐value
	Mean ± SD	Median (25%–75%)	Mean ± SD	Median (25%–75%)	
Mean CIMT (mm)	0.53 ± 0.07	0.52 (0.48–0.57)	0.52 ± 0.04	0.51 (0.49–0.55)	0.96
Plaque presence (Bulb), *N* (%)	2 (7)	NA	3 (10)	NA	1
Maximum plaque height (mm)	1.53 ± 0.49	0 (0, 0)	1.51 ± 0.10	0 (0, 0)	1
Total plaque area (mm^2^)	6.92 ± 3.74	0 (0, 0)	7.71 ± 7.59	0 (0, 0)	0.77
Mean CCA diameter (mm)	5.59 ± 0.39	5.51 (5.33–5.79)	5.42 ± 0.43	5.30 (5.18–5.70)	0.06
Max ICA PSV (cm/s)	91.5 ± 15.5	91.9 (79.0–101.3)	89.3 ± 15.7	91.0 (73.8–102.3)	0.7
Max ICA EDV (cm/s)	29.9 ± 6.21	30.9 (25.3–33.1)	30.2 ± 6.70	30.1 (24.0–34.3)	0.88
Max ICA/CCA PSV ratio	0.93 ± 0.16	0.91 (0.82–1.04)	0.88 ± 0.15	0.88 (0.77–1.13)	0.33

Data presented as mean ± standard deviation.

Group differences assessed by Fisher’s exact test or Wilcoxon–Mann–Whitney two‐sample test.

Abbreviation: CCA, common carotid artery; CIMT, carotid intima‐media thickness; EDV, end‐diastolic velocity; ICA, internal carotid artery; *N*, number; PSV, peak systolic velocity.

**FIGURE 2:**
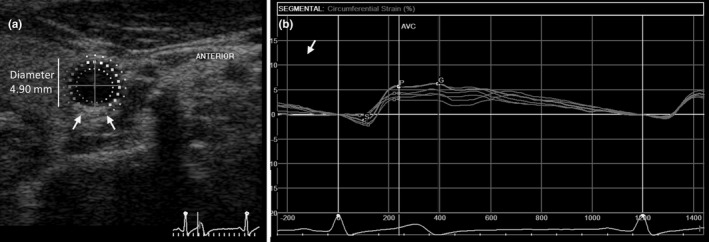
Measurement of far‐wall circumferential strain (FWCS). (a) The region of interest was selected by tracing the common carotid artery wall in short‐axis view. Arrows indicate angular span measured. (b) The FWCS curve is shown on the right. The average peak strain (“G”, indicated by arrow) from three cardiac cycles was used as FWCS value for each carotid (the peak strain value is the most positive value)

Carotid far‐wall circumferential strain (FWCS) was assessed as a measure of vessel stiffness between groups (Figure 2). In comparing the arterial sides, the right‐side FWCS did not differ between PE versus CTRL groups, even after adjustment (index) to vessel diameter, pulse pressure, and heart rate (Table 4). However, FWCS in the left carotid artery was lower in PE women (8.85 ± 2.73 vs. 10.73 ± 3.49; *p* = 0.05) and was significantly decreased after adjustment for vessel diameter (1.61 ± 0.53 vs. 1.98 ± 0.62; *p* = 0.02), pulse pressure (0.230 ± 0.071 vs. 0.295 ± 0.100; *p* = 0.02), heart rate (0.129 ± 0.042 vs. 0.163 ± 0.054; *p* = 0.01), and both pulse pressure and heart rate (3.35 ± 1.08 × 10^−3^ vs. 4.46 ± 1.40 × 10^−3^; *p* = 0.003). Left carotid artery FWCS remained lower in PE women compared to controls after exclusion of all participants meeting IDF criteria for hypertension (6.19 × 10^−4^ vs 8.25 × 10^−4^, *p* = 0.003). Furthermore, left FWCS did not correlate with time postpartum in weeks (rho = 0.13; *p* = 0.35).

**TABLE 4:** Speckle tracking far‐wall circumferential strain of the common carotid arteries

Variable	Preeclampsia (*N* = 30)		Control (*N* = 30)		*p*‐value
	Mean ± SD	Median (25%–75%)	Mean ± SD	Median (25%–75%)	
Right side					
FWCS	10.5 ± 3.51	10.2 (8.33–12.6)	10.3 ± 2.51	10.4 (8.80–11.84)	0.96
FWCS/Diameter	1.89 ± 0.69	1.73 (1.40–2.33)	1.92 ± 0.51	1.91 (1.56–2.32)	0.67
FWCS/PP	0.270 ± 0.083	0.253 (0.222–0.321)	0.282 ± 0.065	0.273 (0.237–0.334)	0.5
FWCS/HR	0.154 ± 0.056	0.142 (0.117–0.183)	0.160 ± 0.051	0.152 (0.129–0.184)	0.55
FWCS/(PP*HR)	3.95 ± 1.24 ×10^−3^	3.61 × 10^−3^ (2.21–4.87 × 10^−3^)	4.34 ± 1.22 ×10^−3^	4.22 × 10^−3^ (3.37–5.12 × 10^−3^)	0.21
(FWCS/Diameter)/(PP*HR)	7.11 ± 2.48 ×10^−4^	6.60 × 10^−4^ (5.45–8.87 × 10^−4^)	8.07 ± 2.43 ×10^−4^	7.79 × 10^−4^ (6.17–9.44 × 10^−4^)	0.08
Left side					
FWCS	8.85 ± 2.73	9.16 (6.35–10.8)	10.7 ± 3.49	10.0 (7.99–13.9)	0.05
FWCS/Diameter	1.61 ± 0.53	1.70 (1.13–1.98)	1.98 ± 0.62	1.89 (1.46–2.48)	0.02*
FWCS/PP	0.230 ± 0.071	0.234 (0.169–0.293)	0.295 ± 0.100	0.291 (0.205–0.360)	0.02*
FWCS/HR	0.129 ± 0.042	0.128 (0.091–0.152)	0.163 ± 0.054	0.158 (0.119–0.203)	0.01*
FWCS/(PP*HR)	3.35 ± 1.08 ×10^−3^	3.20 × 10^−3^ (2.57–4.18 × 10^−3^)	4.46 ± 1.40 ×10^−3^	4.18 × 10^−3^ (3.18–5.35 × 10^−3^)	0.0030*
(FWCS/Diameter)/(PP*HR)	6.11 ± 2.14 ×10^−4^	5.88 × 10^−4^ (4.49–7.40 × 10^−4^)	8.23 ± 2.43 ×10^−4^	7.87 × 10^−4^ (6.23–10.0 × 10^−4^)	0.001*
Mean of both sides					
FWCS	9.69 ± 2.77	9.61 (7.70–11.9)	10.5 ± 2.32	10.7 (8.73–12.1)	0.24
FWCS/Diameter	1.75 ± 0.55	7.72 (2.11)	1.95 ± 0.43	1.98 (1.61–2.25)	0.1
FWCS/PP	0.250 ± 0.068	0.250 (0.200–0.298)	0.288 ± 0.065	0.293 (0.227–0.380)	0.04*
FWCS/HR	0.142 ± 0.045	0.132 (0.109–0.168)	0.162 ± 0.042	0.163 (0.126–0.192)	0.04*
FWCS/(PP*HR)	3.65 ± 1.05 ×10^−3^	3.49 × 10^−3^ (2.90–4.05 × 10^−3^)	4.40 ± 1.01 ×10^−3^	4.33 × 10^−3^ (3.62–5.25 × 10^−3^)	0.005*
(FWCS/Diameter)/(PP*HR)	6.61 ± 2.11 ×10^−4^	6.45 × 10^−4^ (5.18–7.50 × 10^−4^)	8.15 ± 1.81 ×10^−4^	8.36 × 10^−4^ (6.78–9.20 × 10^−4^)	0.002*

Data presented as mean ± standard deviation.

Abbreviations: FWCS, far‐wall circumferential strain; HR heart rate; PP, pulse pressure.

* indicates significant (*p* < 0.05) difference as assessed by Fisher’s exact test or Wilcoxon–Mann–Whitney two‐sample test.

Mean FWCS did not differ between subject groups, but became significantly reduced in PE participants after adjustment for pulse pressure (0.250 ± 0.068 vs. 0.288 ± 0.065; *p* = 0.04); heart rate (0.142 ± 0.045 vs. 0.162 ± 0.042; *p* = 0.04); and pulse pressure and heart rate (3.65 ± 1.05 × 10^−3^ vs. 4.40 ± 1.01 × 10^−3^; *p* = 0.001).

**FIGURE 3:**
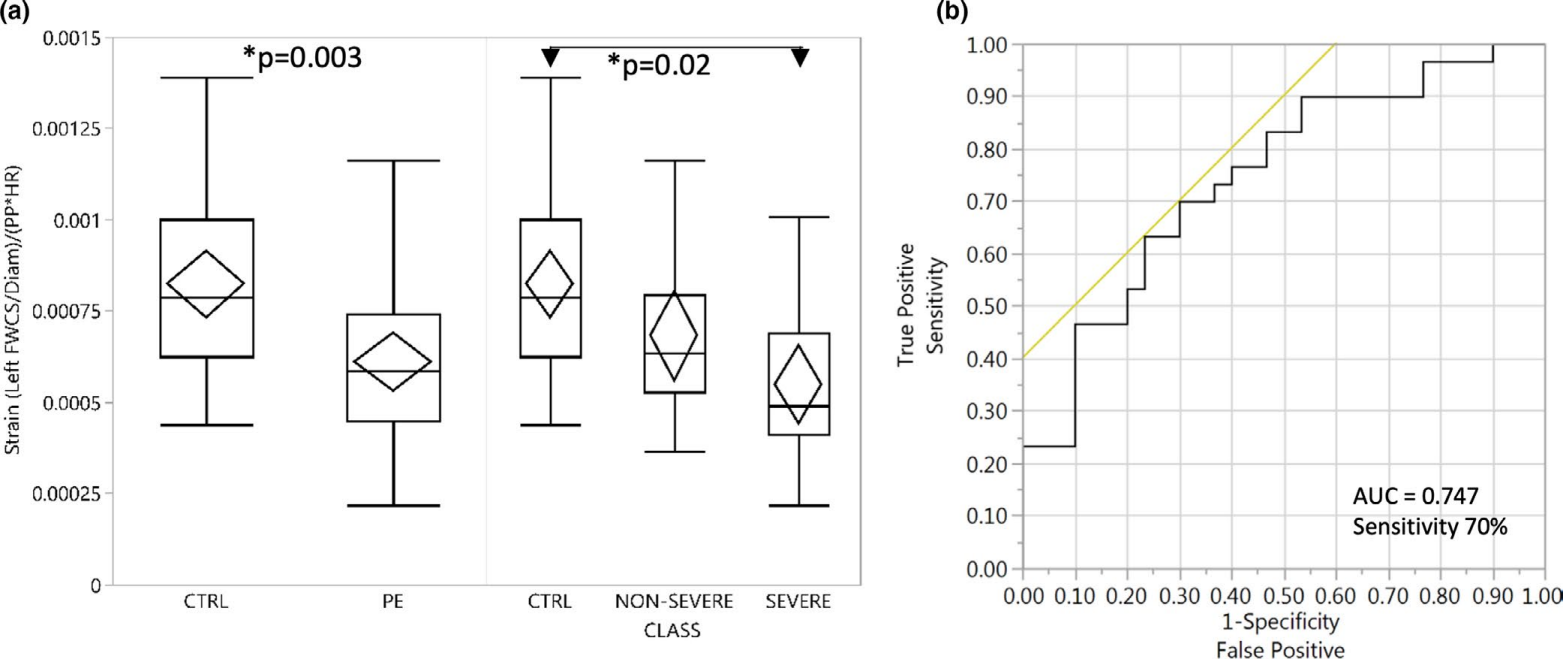
Far‐wall circumferential strain (FWCS) identifies history of preeclampsia. (a) Box plot compact view of distribution with quartiles and outliers of carotid strain variable indicating decreased strain (increased stiffness) in women with a history of preeclampsia (PE) (N=30), in both non‐severe PE (N=14) and severe PE cases (N=16), compared to women with a previous uncomplicated pregnancy (*N* = 30) (*p* = 0.02). The center line in the box shows the median for the data (50th percentile) and the length of the box is the difference between the 75th and the 25th percentiles (interquartile range). The whiskers are the minimum and maximum data values, excluding outliers. *Statistical significance (*p* < 0.05) with Steel–Dwass multiple comparison test compared to control. (b) Receiver operating characteristic area under the curve showing that adjusted left carotid strain has a good sensitivity and specificity for PE

To determine if the degree of PE severity had an effect on FWCS, we stratified FWCS into PE with and without severe features. We looked at the left side and found that PE with severe features had the greatest decrease in FWCS adjusted to diameter, pulse pressure, and heart rate compared to healthy controls (*p* = 0.02) (Figure 3a). CTRL were not significantly different than the non‐severe PE group (*p* = 0.15). The non‐severe and severe groups were not significantly different (*p* = 0.17).

### 3.4 Factors Associated with PE

To assess independent correlations between cardiovascular parameters in relation to women with prior PE, we constructed a logistic regression model through backward selection. Selected co‐variates were the ones found to be significantly different between PE and control participants in Tables 1 and 2. Of all candidate co‐variates, left‐adjusted strain to diameter (Parameter estimates ChiSquare = 6.73; *p* = 0.01) and cholesterol (ChiSquare = 4.65; *p* = 0.03) remained independently associated with PE (Table 5).

**TABLE 5:** Factors associated with PE following stepwise backward selection regression analysis

Term	Estimate	Std Error	Lower 95%	Upper 95%	Chi Square	P>ChiSq
(Left FWCS/Diam)/(PP*HR)	−5240.04	2019.20	−9859.35	−1747.54	6.73	0.01*
Cholesterol (mmol/L)	1.62	0.75	0.41	3.38	4.65	0.03*
Age (years)	−0.28	0.15	−0.63	−0.02	3.35	0.07

Model co‐variates: Age (years), Body mass index (kg/m2), WC (cm), Systolic blood pressure (mmHg), Diastolic blood pressure (mmHg), Ever smoked, *N* (%), Hypertension, *N* (%), Cholesterol (mmol/L), and adjusted left FWCS (Left FWCS/Diameter)/(PP*HR). Only contributors that remained in the model are presented.

Abbreviations: FWCS, far‐wall circumferential strain; HR, heart rate; PP, pulse pressure.

* indicates significant (*p* < 0.05) independent association as assessed with Chi‐Square test.

We also looked at the associations between PE and left‐adjusted FWCS. Without any covariates, PE was significantly associated with adjusted FWCS (Std. Beta = −0.43; *p* = 0.0007). When using logistic regression for all co‐variates (including PE as a co‐variate), only PE was found to be an independent factor associated with left‐adjusted FWCS (Std. Beta = −0.38; *p* = 0.006).

To determine if the adjusted left carotid strain could accurately identify women with a history of PE, we looked at predictive values from a ROC curve. Adjusted left FWCS had a ROC AUC of 0.747 for identifying PE. Using the optimal cut‐off value of 6.99 × 10^−4^, carotid left strain was able to identify formerly PE women with a sensitivity of 70% and a specificity of 70%. The mean adjusted carotid strain of the two sides had a ROC AUC of 0.733 for PE (Figure 3b). Using the optimal cut‐off value of 8.01 × 10^−4^, the mean adjusted carotid strain was able to identify PE with a sensitivity of 83% and a specificity of 63%.

## 4 DISCUSSION

Certain obstetrical complications, like hypertensive disorders of pregnancy, are now widely regarded as indicators of future CVD (Smith et al., 2019). While PE is associated with an increased future risk of CVD, the physiological mechanisms that precipitate this effect are largely unknown. Arterial stiffness is associated with blood pressure dysregulation and incipient atherosclerotic processes (Laurent et al., 2001; Palombo & Kozakova, 2016; Popele et al., 2001). In the present study, we assessed several measures of carotid artery dysfunction using 2D ultrasound in women who had a history of PE or uncomplicated pregnancy.

PE in pregnancy is associated with future atherosclerotic disease and metabolic syndrome (Murphy et al., 2014, 2015; Sabour et al., 2007). This group had a higher BMI and blood pressure, as well as total cholesterol, compared to normotensive women, and was more likely to be defined as hypertensive according to International Diabetes Foundation criteria (Leung et al., 2017). While 8 women in the study totally met IDF criteria for elevated blood pressure, only 1 had blood pressure that would require medication. They were not presently medicated likely due to young maternal age. Nonetheless, the finding of increased carotid artery stiffness after PE was recapitulated after exclusion of all participants with increased blood pressure. PE participants were more likely to have a history of smoking, which opposes existing literature suggesting smoking has a protective effect on PE development (England & Zhang, 2007). However, we did not obtain any information about the frequency nor timing of smoking in relation to the index pregnancy. While other cardiometabolic factors including HDL and LDL did not differ between study groups, other investigations have identified higher blood cholesterol in women with previous PE (He et al., 1999; Manten et al., 2007; Smith et al., 2009). However, increased total cholesterol in formerly PE participants may simply stem from higher average BMI among these women. Carotid plaque area and height are effective surrogates of coronary artery stenosis (Colledanchise et al., 2019; Johri et al., 2016). However, carotid plaque deposition, an atherosclerotic endpoint, is uncommon in women of child‐bearing age. This may explain our finding that PE was not correlated to carotid artery plaque burden. Consistent with its pro‐oxidative, pro‐inflammatory phenotype, a history of PE has been associated with an increased CIMT from as early as 12 months after pregnancy, up to more than 10 years’ post‐partum (Aykas et al., 2015; Garovic et al., 2017; Goynumer et al., 2013). However, McDonald et al. failed to observe a similar difference in their assessment of carotid CIMT in women two decades after PE, which found that the variable did not differ between groups. However, electrocardiogram results in PE women were more often indicative of atherosclerotic processes. This led researchers to conclude that CIMT had been altered by higher antihypertensive and anti‐platelet use by the case group (McDonald et al., 2013). Christensen et al. also did not find any differences in arterial stiffness, operationalized as aortic pulse wave velocity and central augmentation index, between PE and controls, though there were differences between subject groups in terms of cardiovascular risk (Christensen et al., 2016). They concluded Framingham risk may be a predictor of CIMT after PE (Christensen et al., 2016). In our study, between 6 months – 5 years after pregnancy, CIMT and carotid plaque burden were not different between PE and control subjects. This may suggest that additional risk assessment tools are required to adequately capture nascent cardiovascular disease processes.

The most common tool to assess arterial stenosis is vascular Doppler ultrasound. Carotid peak systolic velocity (PSV) and common carotid end diastolic velocity (EDV) are functional variables which serve as potential harbingers of future cerebrovascular disease (Androulakis et al., 1996; Jensen‐Urstad et al., 1999; Lloyd et al., 2012; Saba et al., 2016; Strosberg et al., 2017; Yang et al., 2014). We did not find significant differences in Doppler measurement between the groups and all measures were considered normal. Our present findings suggest that PSV and EDV are not appreciably modulated following a pregnancy complicated by PE.

Carotid FWCS is a novel measure which was initially adapted from a similar computational method for assessing cardiomyocyte function (Yang et al., 2011). FWCS is directly associated with both CVD markers, including CIMT, plaque, and shear stress in hypertensive patients, and adverse cardiovascular outcomes such as diabetes and hypertension (Patton et al., 2018; Saito et al., 2012; Yang et al., 2014). Previous work by our group has positioned FWCS as a reliable marker of sub‐clinical atherosclerotic processes (Patton et al., 2018). In the present study, we have observed that left carotid FWCS is lower (increased arterial stiffness) in subjects with prior PE, with a greater decrease associated with individuals with a history of severe PE. Logistic modelling of these data show left carotid FWCS and total serum cholesterol to be independently associated with diagnosis of PE—not participants’ blood pressure, BMI, age, and 30‐year cardiovascular risk score. While data on left carotid FWCS is novel, association of total serum cholesterol with PE is congruent with existing literature which suggests women have an adverse cardiometabolic profile after the complication (Manten et al., 2007; Smith et al., 2009). These findings suggest that left carotid FWCS and serum cholesterol are linked to the maternal milieu that either leads to, or results from, a pregnancy complicated by PE. Interpreting this is a matter of philosophy. PE can be understood as an indicator of existing sub‐clinical dysfunction, or an independent event which permanently modulates cardiovascular risk (Chen et al., 2014; Craici et al., 2008; Newstead et al., 2007). Regardless of the physiological origin, these findings corroborate existing evidence that a history of PE is associated with sub‐clinical differences in cardiovascular risk profile that may be linked to adverse later‐life outcomes (McDonald et al., 2008).

While carotid FWCS has not been previously examined in PE populations, increased arterial stiffness is associated with PE development, and in limited studies has been shown to be elevated shortly after delivery, persisting up to 18 months postpartum (Kaihura et al., 2009; Robb et al., 2009; Torrado et al., 2015; Yuan et al., 2013). While the precise mechanisms are unknown, endothelial dysfunction associated with PE includes leukocyte adhesion, oxidative stress, and decreased vascular nitric oxide bioavailability (Holthe et al., 2004; Hubel, 1999; Sandrim et al., 2010). It stands to reason that these nascent atherosclerotic mechanisms may provoke permanent elasticity changes in large vessels, as is already known to occur in atherosclerosis (Cohn, 2004). The degree of endothelial dysregulation in PE with severe features corroborates our observations of increased stiffness associated with severe disease.

The asymmetry of our FWCS results, namely, lower strain, and thicker CIMT in the left carotid artery, has been reported before, though some have questioned this finding (Luo et al., 2011; Yang et al., 2011; Yuda et al., 2011). Arterial distensibility changes in atherosclerosis are more likely to occur in areas with turbulent flow (Davies et al., 1986). Therefore, atherosclerotic processes are more likely to occur in the left common carotid artery, which is a direct branch of the aorta, compared to the right common carotid artery, which is a branch of the brachiocephalic artery. Strain reflects muscle fiber composition, orientation, and shortening in three dimensions. Thus, it is possible that the increased medial thickness suggests differences in smooth muscle parameters between the left and right carotid artery. We speculate that the increased stiffness observed in pre‐eclamptic women on the left compared to the right may reflect a greater musculature of this vessel. Additionally, carotid hemodynamic asymmetry lends to a greater incidence of left‐side stroke (Hedna et al., 2013; Hernández et al., 2003). Further investigation in a larger cohort is needed.

Finally, ROC analyses of the present data identify mean‐adjusted FWCS as a good indicator of PE history with a sensitivity of 83%. Interpretation of these results from a physiological standpoint is difficult, owing to ongoing debate about whether downstream health outcomes of PE are due to the disease per se or pre‐existing dysfunction which precipitated the condition. If the latter is true, then differences in FWCS could possibly be evident in women at higher risk for developing PE. Therefore, future exploratory studies should examine the association of FWCS in predicting pregnancy PE development, to evaluate its utility as a clinical risk stratification tool. FWCS surveillance in pregnancy could occur alongside ultrasonographic appointments for dating (11–14 weeks) and fetal anatomy (18–20 weeks) in the first and second trimesters.

Some limitations of our study require discussion. Our PE group had a significantly higher BMI and were younger in age compared to the uncomplicated control group. Additionally, convenience sampling among control participants introduces a degree of bias and may hamper vascular results in this cohort from being broadly generalizable. Since our organization is a tertiary hospital, our cases were more likely to have severe features, thus potentially posing a referral bias. A higher representation of severe‐feature PE in this study may not accurately represent the typical course of vascular function after PE. While 8 women had elevated blood pressure (and 1 was technically eligible to be medicated), our results were unchanged after exclusion of these participants. Additionally, hypertension was accounted for in our stepwise logistic regression model and found not to be a significant covariate. Owing to our inclusion of only one time point in the study protocol, we do not have information on participants’ vascular risk prior to pregnancy or PE. PE development in pregnancy may represent a failure of the maternal vasculature to adapt to the stressors of advancing gestation (Smith et al., 2019). Therefore, we cannot confirm if PE is the cause of decreased strain or if this observation is due to a predisposition in these women prior to pregnancy. Future work is needed to follow women prior to pregnancy and assess changes in strain postpartum in relation to development of PE.

Risk stratification after PE and the identification of associated mechanisms which contribute to future disease are essential improvements to clinical management following PE in pregnancy. We conclude that our findings suggest carotid left FWCS is decreased after PE in a severity‐dependent manner. These findings are congruent with the growing clinical understanding of PE as an independent risk factor for downstream cardiovascular disease. Future efforts should be aimed at identifying contributing mechanisms to cardiovascular risk after PE that are amenable to therapeutic or lifestyle intervention.

## CONFLICT OF INTEREST

None declared.

## AUTHOR CONTRIBUTIONS

MFH, AJ, LB, and GS developed the carotid protocol; LB conducted recruiting; JH conducted participant visits; MFH and LB conducted statistical analyses; LB prepared written manuscript, which was edited by MFH, AJ, JH, and GS.
